# Effects of ramie at various levels on ruminal fermentation and rumen microbiota of goats

**DOI:** 10.1002/fsn3.1449

**Published:** 2020-02-10

**Authors:** Encun Du, Wanzheng Guo, Fang Chen, Qiwen Fan, Na Zhao, Wei Zhang, Shaowen Huang, Jintao Wei

**Affiliations:** ^1^ Hubei Key Laboratory of Animal Embryo and Molecular Breeding Institute of Animal Science and Veterinary Medicine Hubei Academy of Agricultural Sciences Wuhan China

**Keywords:** goat, ramie, rumen microbiota, ruminal fermentation

## Abstract

With the shortage of high‐quality forage in southern China, it is urgent to develop local unconventional forage resources, such as ramie. The objective of this study was to investigate the effects of dietary ramie levels on ruminal fermentation and rumen microbiota of Boer goats. A total of 60 Boer kids were allocated into four dietary treatments with 0, 10%, 20%, and 40% ramie, respectively. The results showed that the ruminal fermentation parameters were unaffected by the inclusion of 0%–20% ramie (*p* > .05). However, the ruminal concentration of total short‐chain fatty acids, acetate, and butyrate increased linearly with increasing ramie proportions (*p* < .05). Notably, ramie at 40% level improved the production of butyrate compared with the other dietary treatments (*p* < .05). Increasing the dietary ramie proportion did not affect the alpha or beta diversity of the rumen microbial community, and the relative abundances of the microorganisms at phylum level and most of the identified microorganisms at genus level remain unchanged (*p* > .10) even though the relative abundance of *Asteroleplasma* and *Treponema* was increased respectively when 10% and 20% ramie was included (*p* < .05). Overall, the result of this study demonstrated that up to 40% ramie had no impairment in the ruminal fermentation or rumen microbiota of goats.

## Graphical abstract

With the shortage of high‐quality forage in southern China, it is urgent to develop local unconventional forage resources, such as ramie. The current study with goats showed that the ruminal concentration of total short‐chain fatty acids, acetate, and butyrate increased linearly with increasing ramie proportions. The relative abundances of the microorganisms at phylum level and most of the identified microorganisms at genus level remain unchanged, which demonstrated that up to 40% ramie had no impairment in the ruminal fermentation or rumen microbiota of goats.

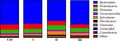

## 1 INTRODUCTION

Ramie (*Boehmeria nivea*) is a perennial plant in the nettle family of *Urticaceae*, which is native to China and internationally known as *China grass*. Ramie has been grown as fiber crop in China for a long time, and the fiber production of ramie in China is 500,000 ton per year, contributing more than 96% of global ramie fiber production (Kipriotis, Heping, Vafeiadakis, Kiprioti, & Alexopoulou, 2015). However, recent studies showed that ramie is a good source of nutritious green biomass, palatable to domestic livestock (Li et al., 2018; Zhang et al., 2019). There is a long‐term shortage of high‐quality forage in southern China, which challenges the development of ruminant breeding. The high temperature and high humidity climatic conditions and acidic soil conditions are extremely detrimental to the growth of alfalfa, but it is conducive to the growth of ramie (Wei et al., 2018). Ramie can be harvested up to 7 times per year and can grow to 1.0 m during each harvesting period in Hubei province (Wei et al., 2018).

In addition to high production, the nutritional composition of ramie leaves was similar to that of alfalfa. According to our previous study, the crude protein, lysine, and threonine content of ramie are 19.4%, 0.91%, and 0.87%, respectively, and the content of neutral detergent fiber (NDF) and acid detergent fiber (ADF) are moderate (Wei et al., 2019). Therefore, ramie could act as a high‐quality forage resource in southern China.

We have conducted a study to evaluate the effects of dietary ramie levels (0, 10%, 20%, and 40%) on the growth performance, serum biochemical indices, and meat quality of Boer goats (Wei et al., 2019). Results showed that the average daily gain and feed/gain ratio were not influenced by the inclusion of 0%–40% ramie, but average daily feed intake (dry matter intake) was significantly decreased when 40% ramie was included (Wei et al., 2019). In addition, the ramie inclusion had no adverse effect on the health or meat quality of goats (Wei et al., 2019). However, as a new type of protein ingredient for ruminants, less information is available documenting the effects of ramie on ruminal fermentation, rumen microbial composition or structure.

Therefore, the objective of the present trial was to investigate the effects of dietary ramie levels on the ruminal fermentation parameters and rumen microbiota of Boer goats, which was also a continuation of our previous work (Wei et al., 2019).

## 2 MATERIALS AND METHODS

### 2.1 Ramie

The ramie variety “E‐Mu‐Zhu 0904” bred by the Hubei Academy of Agricultural Sciences and Xianning Academy of Agricultural Sciences was used in the present study. The plants were grown at the Ramie Demonstration Base of the Xianning Academy of Agricultural Sciences (113°32′E to 114°58′E and 29°02′N to 30°19′N). The ramie plants were harvested when they reached a height of 0.8–1.0 m, and roots were retained. After the harvest, plants were chopped to 2‐cm pieces and air‐dried. The nutritional composition of ramie hay was 90.74% dry matter (DM) and 19.43% crude protein, 3.46% ether extract, 49.81% NDF, 43.26% ADF, and 15.17% crude ash based on DM.

### 2.2 Animals, diets, and experimental design

A total of 60 Boer kids (3‐month‐old; half male and half female; average bodyweight = 19.72 ± 2.53 kg) were used in the 90‐day feeding experiment. The trial was conducted at Tianyue Animal Husbandry Company in Hubei Province. The total experimental period comprised 90 days, including a 15‐day pretest and a 75‐day formal test. The goats were randomly divided into four groups (Ctrl, I, II, and III) and fed diets with 0, 10%, 20%, and 40% ramie, respectively. The diets were processed into pellets with a particle size of 5 mm using a flat diet pelletizer (DL‐150). The composition of the diets and nutritional values are listed in Table 1.

**Table 1:** Diet composition and nutrient levels (based on air‐dried material)

Item (%, unless otherwise indicated)	Ctrl	I	II	III
Corn	32.19	33.57	35.10	39.45
Soybean meal	14.21	11.53	8.90	3.60
Peanut vine	50.00	41.40	32.50	13.60
Ramie	0.00	10.00	20.00	40.00
Dicalcium phosphate	2.20	2.10	2.10	1.95
Salt	0.40	0.40	0.40	0.40
Premix^a^	1.00	1.00	1.00	1.00
Nutrient levels^b^				
Digestible energy (MJ/kg)	11.30	11.30	11.30	11.30
Crude protein	14.50	14.50	14.50	14.50
Calcium	1.76	1.87	1.99	2.19
Total phosphorus	0.61	0.61	0.61	0.61
Neutral detergent fiber	31.13	31.15	31.11	30.80
Acid detergent fiber	22.98	23.16	23.29	23.35

a The premix provided the following (per kilogram of diet): iron, 80 mg; copper, 10 mg; zinc, 50 mg; manganese, 30 mg; selenium, 0.30 mg; iodine, 0.80 mg; cobalt, 0.80 mg; vitamin A, 10,000 IU; vitamin D_3_, 3,000 IU; vitamin E, 50 mg.

b Calculated value for digestible energy, measured values for the rest.

All goats were individually housed in pens and the ambient temperature range was 15°C to 25°C during the experiment. The goats were fed twice daily at 08:00 and 17:00 and had access to feed and water ad libitum.

### 2.3 Sampling

At the end of the experiment, goats were fasted for 24 hr before slaughter. Then, six goats with similar body weight were randomly selected and sacrificed. Ruminal content samples were collected into two sterile 50‐ml plastic containers in a sterile manner immediately after the slaughter. Then, all the ruminal contents were squeezed through four layers of cheesecloth to remove particulate matter. One copy of the remaining ruminal fluid was stored at −80°C for DNA extraction. Two drops of saturated mercury dichloride (HgCl_2_) solution were added to the other copy of the remaining ruminal fluid to inactivate the rumen microorganisms, and supernatant was collected after centrifugation at 15,000 *g*/min at 4°C for 15 min. The supernatant was stored at −20°C until analysis.

### 2.4 Ruminal fermentation parameters

The pH value of the supernatant was determined with a pH meter. The ammonia nitrogen content was analyzed with the method of phenol‐sodium hypochlorite colorimetric (Broderick & Kang, 1980). For short‐chain fatty acid (SCFA) analysis, gas chromatography was applied using Dionex ICS‐3000 ion chromatograph, and the analytical column was IonPac AS‐HC separation column (4 mm × 250 mm) with IonPac AG 11‐HC guard column (4 mm × 50 mm).

### 2.5 DNA extraction, PCR amplification of 16S rRNA gene, and sequencing

16S rRNA gene of rumen microbiota was sequenced and analyzed as described by Yin et al. (2017). Briefly, microbial genomic DNA was extracted from the ruminal samples using the QIAamp DNA Stool Mini Kit (Qiagen Inc.) according to the manufacturer's instructions. Then, the concentration of DNA was determined using spectrophotometry and its quality was evaluated using 2% agarose gel electrophoresis. The microbial 16S rRNA gene was amplified using the universal primer sets 515F (5′‐GTGCCAGCMGCCGCGGTAA‐3′) and 806R (5′‐GGACTACHVGGGTWTCTAAT‐3′) with a unique error‐correcting barcode for each sample. The barcoded amplicons were visualized using 2% agarose gel electrophoresis and purified using the AxyPrep DNA Gel Extraction kit (Axygen Inc.). The purified amplicons were pooled in equal concentrations and sequenced using the pair‐end method on Illumina Miseq250 platform.

For 16S rRNA gene analyzing, the obtained raw reads were firstly merged into sequences based on the relationship among their overlaps, and poor‐ or low‐quality sequences were discarded. Then, the obtained sequences were aligned into operational taxonomic units (OTUs) analysis using the software VSEARCH (version 1.9.6) based on 97% sequence similarity. To display the common and unique OTUs in the four groups, Venn diagram was drawn by software R (version 3.1.1). The rarefaction curve, alpha diversity, and beta diversity were analyzed using QIIME (version 1.7.0). The rarefaction curve showed the sequencing depth. The alpha diversity was represented by the Chao1 and Ace indexes, which characterize the community richness, and the Shannon and Simpson indexes that indicating the community evenness. The principal coordinate analysis (PCoA) based on weighted unifrac distance was performed to represent the beta diversity, which illuminate the species complexity of ruminal bacterial community. The representative sequences were then compared with the RDP database (the Ribosomal Database Project, https://rdp.cme.msu.edu/) using RDP Classifier for taxonomic classification (at 80% confidence threshold) at the kingdom, phylum, class, order, family, and genus levels. It should be noted that two DNA samples of group III were accidently lost before PCR amplification, so results of four replicates were obtained for group III, and six for the other groups.

### 2.6 Statistical analysis

Data of the ruminal fermentation parameters and the alpha diversity of the rumen microbiota were analyzed using one‐way ANOVA and LSD multiple comparison test by SPSS 21.0 software (IBM Inc.). The linear and quadratic effects of dietary ramie levels were also studied using polynomial contracts. Metastats (http://metastats.cbcb.umd.edu/) and R (v3.1.1) were used to determine the difference of rumen microbial composition. Results were expressed as treatment means and pooled *SEM*. Differences were considered significant at *p* ≤ .05, and .05 < *p* < .10 was considered a trend toward significance.

## 3 RESULTS

### 3.1 Ruminal fermentation

According to the current study, goats in each treatment group maintained normal pH value of ruminal fluid (5.5–7.5), and dietary ramie inclusion did not influence the pH value compared with group Ctrl (*p* > .05, Table 2). Notably, goats in group III showed significantly decreased ruminal pH value in comparison with group I (*p* < .05). No significant difference was found among the four treatment groups in the ruminal concentration of ammonia nitrogen (*p* = .128) though a linear upward trend was shown with increasing ramie levels (*p* = .098). The composition of SCFA as well as the ratio of acetate/propionate was similar among groups Ctrl, I, and II (*p* > .05, Table 3). However, the concentration of total SCFA and butyrate increased both linearly and quadratically with increasing ramie proportions (*p* < .05). Also, the concentration of acetate and valerate was increased linearly and quadratically (*p* = .040 and *p* = .043, respectively) by the increase in dietary ramie levels. In addition, goats in group III produced more acetate than group I (*p* < .05), more butyrate in contrast with groups Ctrl, I, and II (*p* < .05), and higher total SCFA compared with groups I and II (*p* < .05).

**Table 2:** Effects of dietary ramie levels on the ruminal fermentation of goats

Item†	Ctrl	I	II	III	*SEM*	*p* value		
						Treatment	Linear	Quadratic
Ruminal pH	7.00^ab^	7.21^a^	7.08^ab^	6.93^b^	0.04	.067	.164	.059
NH_3_‐N (mg/100 ml)	28.28	18.75	24.61	30.34	1.88	.128	.098	.156
Total SCFA (mmol/L)	41.72^ab^	35.08^b^	36.57^b^	54.43^a^	2.82	.050	.041	.047
SCFA profile (mmol/L)								
Acetate	23.09^ab^	19.64^b^	21.44^ab^	30.76^a^	1.65	.075	.040	.094
Propionate	8.41	7.56	7.28	10.02	0.64	.451	.308	.216
Butyrate	4.64^b^	3.39^b^	3.29^b^	7.65^a^	0.51	.002	.003	.004
Isobutyrate	1.98	1.74	1.69	1.97	0.13	.833	.918	.367
Valerate	0.97	0.75	0.70	1.12	0.07	.160	.309	.043
Isovalerate	2.64	2.00	2.16	2.92	0.19	.291	.374	.103
Acetate/propionate	2.83	2.75	3.02	3.11	0.10	.581	.236	.944

Abbreviations: NH_3_‐N, ammonia nitrogen; SCFA, volatile fatty acid; *SEM*, pooled standard error.

^†^ Means not sharing a common superscript letter within the same row differ significantly (*p* < .05).

**Table 3:** Alpha diversity of the rumen microbial community of goats

Item^a^	Ctrl	I	II	III	*SEM*	*p* value		
						Treatment	Linear	Quadratic
Chao1	644.38	725.12	714.16	755.60	23.94	.451	.178	.583
Ace	661.54	746.18	731.62	770.10	24.21	.462	.200	.553
Shannon	4.18	4.62	4.55	4.68	0.09	.196	.100	.307
Simpson	0.06	0.03	0.04	0.03	0.01	.243	.158	.262

a SEM, pooled standard error.

### 3.2 Rumen microbiota

**Figure 1:**
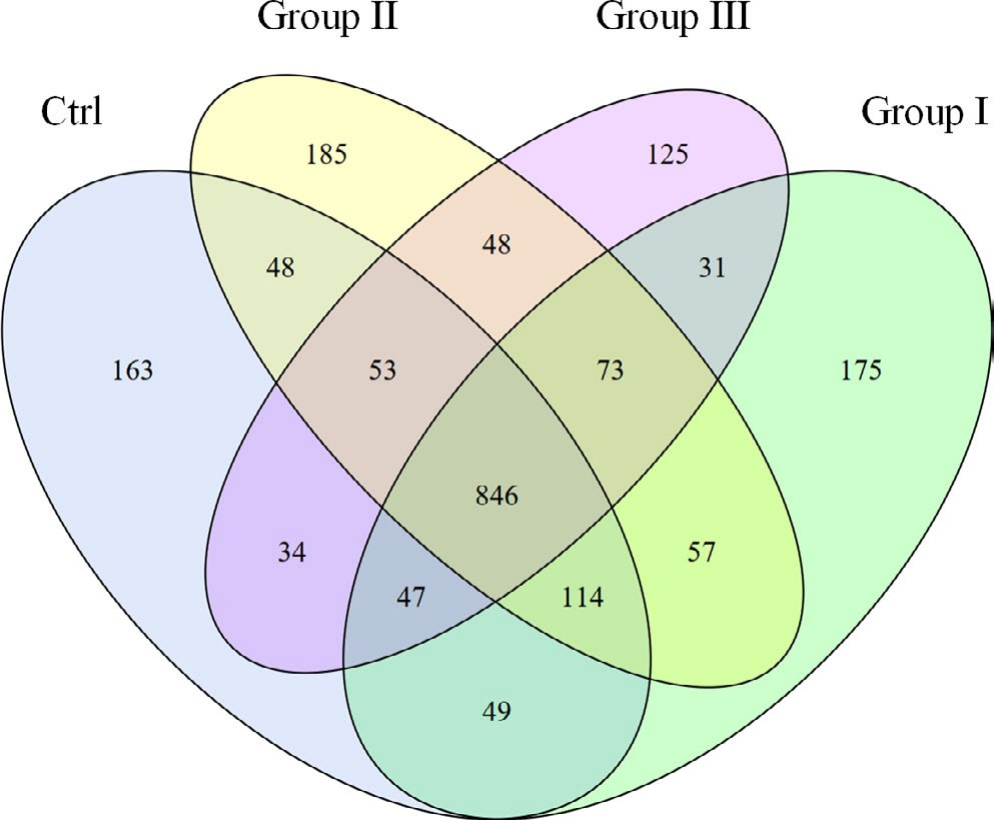
OTU Venn diagram of the rumen microbiota of goats

**Figure 2:**
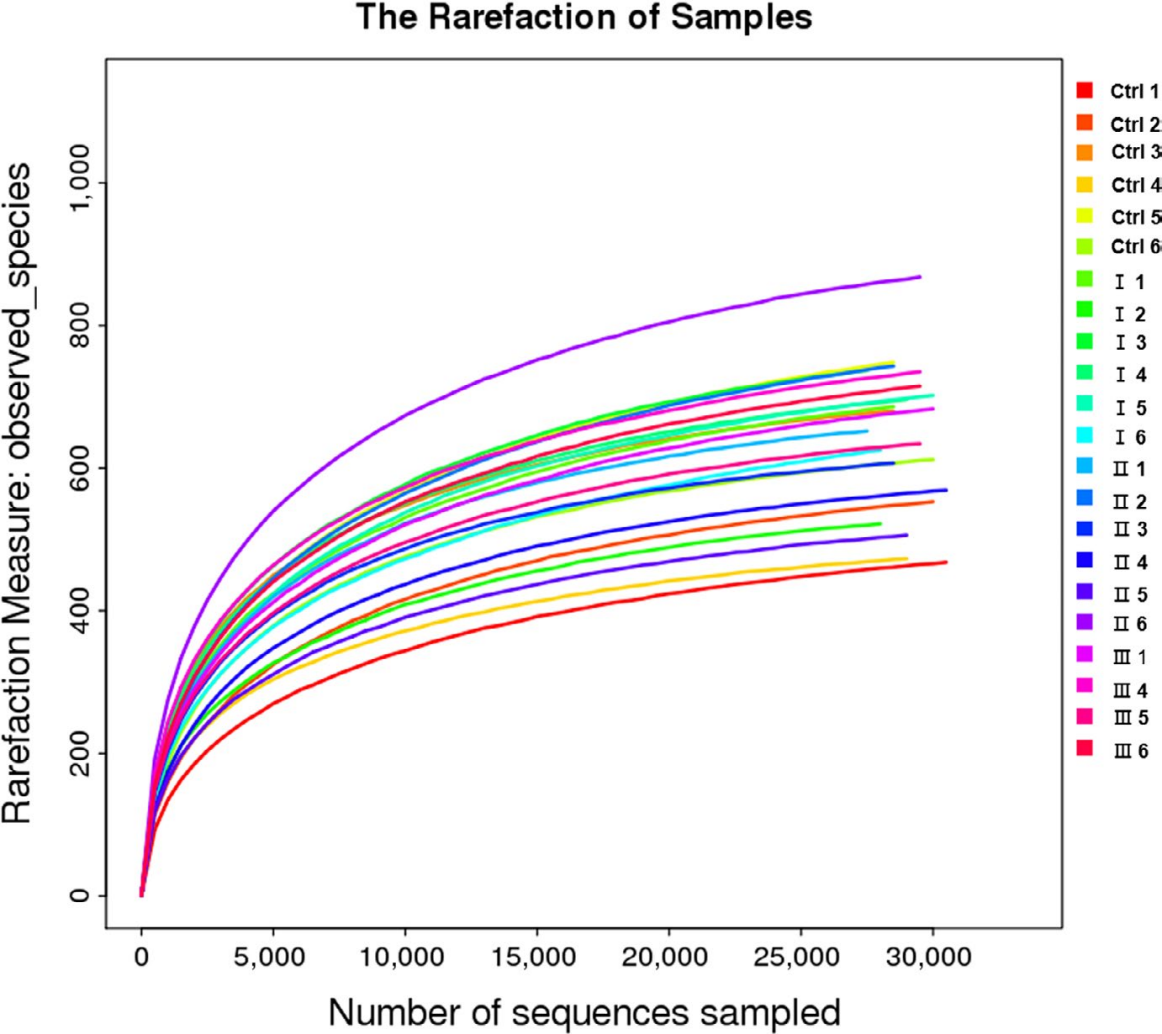
Rarefaction analysis of 16S rRNA gene sequences from the rumen microbiota of goats

In the present study, an average of 43,014 raw reads was obtained from the rumen microbiota, and an average of 39,030 clean reads was remained. According to the OTU Venn diagram (Figure 1), 846 OTUs were shared within the four treatment groups, and 163, 175, 185, and 125 OTUs were unique to groups Ctrl, I, II, and III, respectively. The rarefaction curves of all the samples were nearly asymptotic (Figure 2), indicating that the depth of sequencing covered most of the microorganisms in the sample.

**Figure 3:**
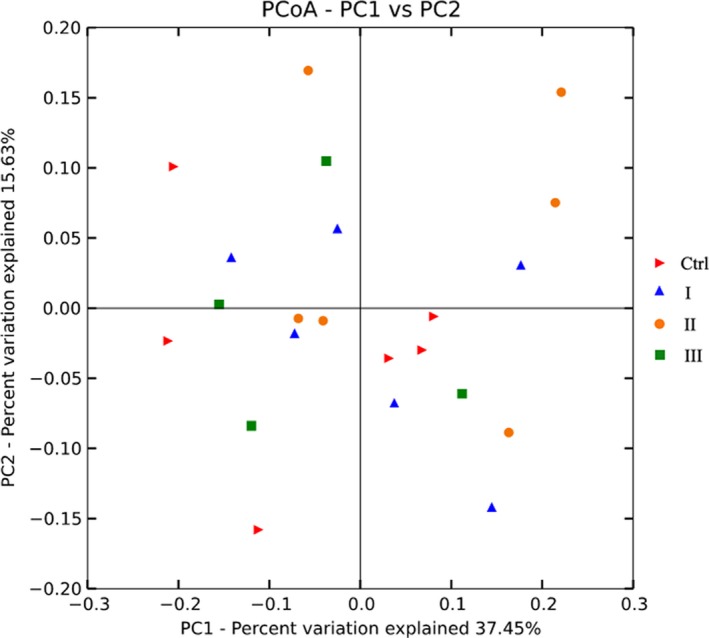
Principal coordinate analysis (PCoA) based on the weighted unifrac distance of the rumen microbial community

In regard to the alpha diversity, no difference in community richness or evenness was observed sine none of Chao1, Ace, Shannon, or Simpson indexes was different among the four treatment groups (*p* > .10, Table 3). According to the PCoA, the four groups clustered together, which illuminated the similar rumen microbial community despite the difference in dietary ramie levels (Figure 3). The principal coordinates 1 and 2 accounted for 37.45% and 15.63% of the total variation, respectively.

**Figure 4:**
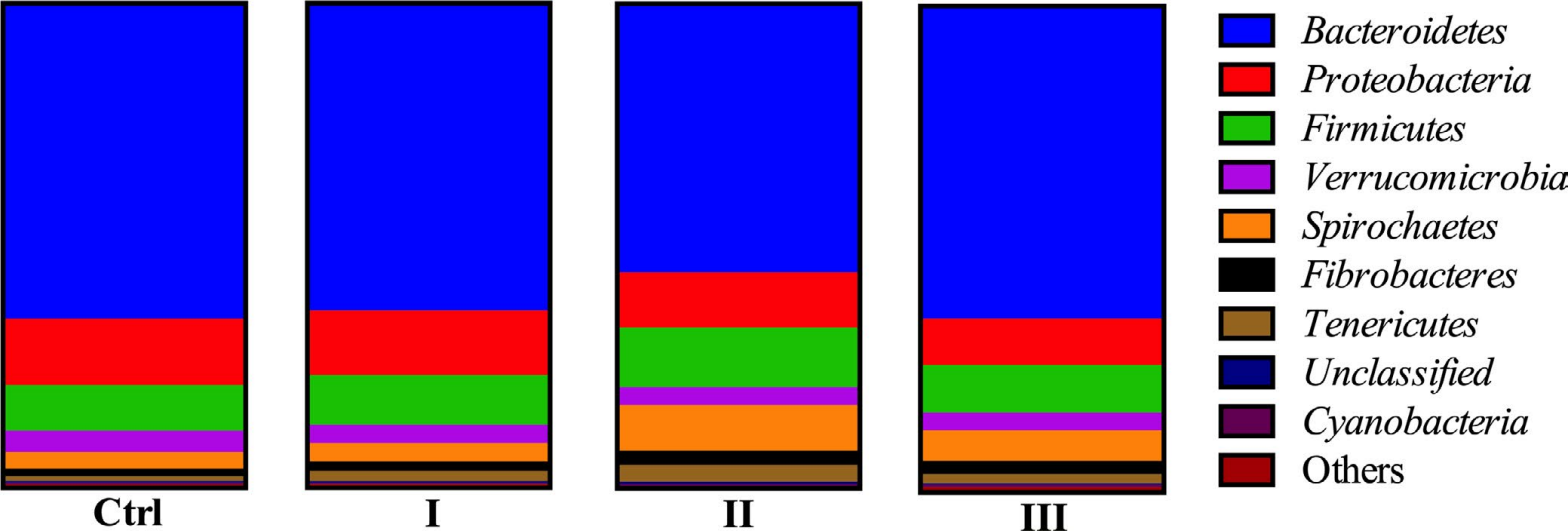
Composition of rumen microbial communities and relative abundances at phylum level

*Bacteroidetes* was the predominant phylum, representing 55%–65% of all sequences, followed by *Proteobacteria*, *Firmicutes*, *Verrucomicrobia*, and *Spirochaetes* (Figure 4). Increasing the ramie proportion from 0% to 40% did not influence the relative abundances of rumen microbiota at phylum level (*p* > .10), though 20% ramie supplementation decreased the relative abundance of *Bacteroidetes* and increased that of *Spirochaetes* numerically.

A total of 69 known genera were identified from the 16S rRNA gene sequences, and there were 62 shared within the Ctrl group, and 56, 59, and 53 shared within groups I, II, and III, respectively. *Prevotella* was the most abundant shared genus, representing 29%–42% of all sequences, which was far greater than those of the other shared genera (Table 4). Dietary ramie levels had no influence on the relative abundances of most rumen microbiota at genus level (*p* > .10), except for *Treponema* and *Asteroleplasma*. In contrast to group Ctrl and group I, goats in group II showed significantly higher abundance of *Treponema* (*p* < .05). Besides, the proportion of *Asteroleplasma* was found greater in the rumen microbiota of group I as compared with group Ctrl and group II (*p* < .05). Notably, a considerable amount of sequences (40.26%–45.73%) was not classified into any genus.

**Table 4:** Composition of rumen microbial communities and relative abundances at genus level

Item (%)†	Ctrl	I	II	III	*SEM*	*p* value
*Bacteroidetes‐Prevotella*	41.88	36.69	29.49	40.24	9.24	.236
*Proteobacteria‐Succinivibrio*	2.95	1.74	1.60	0.21	2.77	.291
*Spirochaetes‐Treponema*	2.89^b^	3.02^b^	8.54^a^	5.72^ab^	4.50	.094
*Fibrobacteres‐Fibrobacter*	1.56	1.99	2.93	2.75	1.70	.375
*Proteobacteria‐Ruminobacter*	1.36	2.12	0.69	1.23	1.55	.177
*Firmicutes‐Succiniclasticum*	1.31	0.89	0.99	1.16	0.63	.649
*Bacteroidetes‐*CF231	1.24	1.98	1.34	1.87	0.86	.362
*Firmicutes‐Oscillospira*	0.81	0.13	0.45	0.09	0.72	.255
*Bacteroidetes‐*YRC22	0.57	0.81	0.60	1.23	0.53	.215
*Tenericutes‐*RFN20	0.50	1.02	1.29	0.49	1.07	.565
*Spirochaetes‐Sphaerochaeta*	0.49	0.66	0.79	0.48	0.51	.385
*Firmicutes‐Ruminococcus*	0.40	0.89	0.46	0.36	0.42	.220
*Firmicutes‐Butyrivibrio*	0.36	0.54	0.49	0.45	0.27	.584
*Firmicutes‐*02d06	0.36	0.25	0.42	0.55	0.27	.227
*Tenericutes‐Anaeroplasma*	0.33	1.05	1.44	0.53	1.16	.114
*Firmicutes‐Roseburia*	0.29	0.18	0.50	0.06	0.58	.567
*Firmicutes‐Clostridium*	0.23	0.28	0.42	0.31	0.23	.509
*Firmicutes‐Schwartzia*	0.18	0.30	0.25	0.19	0.22	.730
*Firmicutes‐Coprococcus*	0.12	0.19	0.30	0.19	0.18	.422
*Firmicutes‐Asteroleplasma*	0.02^b^	0.35^a^	0.10^b^	0.15^ab^	0.04	.004
Others (<0.5%)	0.72	0.76	0.83	1.11	0.43	.560
Unclassified	41.23	43.86	45.73	40.26	7.87	.724

Abbreviation: *SEM*, pooled standard error.

^†^ Means not sharing a common superscript letter within the same row differ significantly (*p* < .05).

## 4 DISCUSSION

Generally, the ruminal concentration of ammonia nitrogen is a reflection of the balance statues of protein degradation and microbial protein synthesis (Santoso, Kilmaskossu, & Sambodo, 2007). According to McDonald et al. (2002), the optimal concentration of ruminal ammonia nitrogen to synthesize microbial protein was 6–21 mmol/L, that is, 8.4–29.4 mg/100 ml. Increasing the dietary ramie levels from 0% to 20% remained the optimal ammonia nitrogen concentration, although 40% ramie proportion resulted in a slightly higher value (30.34 mg/100 ml). 35.42 mg/100 ml of ruminal ammonia nitrogen was found when 36% ramie hay was included in the diet of Liuyang black castrated goats (Zhang et al., 2019), implying possible decreased nitrogen utilization with more than 36% ramie proportion.

Ruminal pH is a key determinant of the profile of nutrients available for absorption. According to the present study, the ruminal pH value of goats in the four dietary treatment groups was maintained between 6.93 and 7.21, which would benefit the bacterial activity of fiber decomposition and protein synthesis in the rumen (Enemark, 2008; Morgante et al., 2009). Studies suggest that dietary carbohydrate source influences ruminal pH (Jurkovich et al., 2014; Khorasani & Kennelly, 2001). In the current study, as the level of ramie increased, the dietary concentrate to forage ratio decreased slightly (from 1.00 to 0.87). Correspondingly, the ruminal pH showed a slight increase in value with increasing ramie levels, but was decreased when 40% ramie was included, exhibiting a tendency of quadratic response to ramie levels. Actually, ruminal pH is in relation to the production, absorption, and flow rate of SCFA and ammonia, and the secretion of buffers into the rumen (Dijkstra et al., 2012). The decreased ruminal pH found in 40% ramie level may be related to the enhanced SCFA yield, which will be discussed later.

Increasing dietary ramie levels from 0% to 20% did not affect the concentration of total SCFA and the profile of SCFA, which indicated that the ruminal fermentation pattern of the diets was unaffected by ramie inclusion below 20%. However, the concentration of acetate, butyrate, and total SCFA was increased linearly by the increase in dietary ramie levels. When 40% ramie was included, the ability of ruminal fermentation was improved significantly as higher concentration of acetate, butyrate, and total SCFA was observed. This result was in accordance with the decreased pH value in the 40% ramie group, supporting the view that ruminal pH is negatively related to the concentration of ruminal total SCFA (Dijkstra et al., 2012). The increased molar concentrations of acetate and butyrate at 40% ramie level could be associated with changes in carbohydrate source. Generally, diets with high neutral detergent soluble fiber (e.g., pectin) increases acetate and butyrate molar concentration (Asadollahi, Ponnampalam, Sari, & Erfanimajd, 2018; Bang et al., 2018). Ramie is rich in pectin (about 5%), and actually the removal of pectin is an important step to make the ramie fiber suitable for textile use (Bhattacharya & Shah, 2002). The effects of pectin contributed by ramie on SCFA molar concentration in rumen fluid could also be attributed to the increased activity of cellulolytic bacteria. It was estimated that SCFA was the major energy source for ruminants and contribute approximately 70% to the caloric requirements of goat, sheep, and cattle (Bergman, 1990). The increased SCFA concentration could partially explain the relatively unchanged average daily gain despite reduced feed intake when 40% ramie was included (Wei et al., 2019).

Ammonia and SCFA are end products of rumen microbial fermentation, and diet had great influence on the microbial community of rumen (Khiaosa‐ard & Zebeli, 2014). Although a few investigations have evaluated the nutritional properties of ramie used as forage resource for livestock (Li et al., 2018; Zhang et al., 2019), less information is available on the rumen microbiota. In the present study, it was found that dietary ramie levels did not affect the alpha and beta diversity of the rumen microbial community. *Bacteroidetes* was the predominant phylum of all 16S rRNA gene sequences of the rumen microbiota, followed by *Proteobacteria*, *Firmicutes*, *Verrucomicrobia*, and *Spirochaetes*, and increasing the ramie levels did not influence the relative abundances of the microbiota at phylum level, which showed the stability of the rumen microbial community. In addition, dietary ramie levels had no effect on most of the identified genera, including the most abundant genera *Prevotella*. However, 20% ramie proportion significantly increased the relative abundance of *Treponema*. It was reported that *Treponema* was closely associated with pectin‐rich diet due to the pectin‐degradation ability of species in this genus (Liu, Pu, Xie, Wang, & Liu, 2015; Yang et al., 2018). As mentioned earlier, ramie is rich in pectin (Bhattacharya & Shah, 2002). Therefore, the increase in the relative abundance of *Treponema* in the present study was a response to the ramie inclusion and may improve the digestibility of ramie pectin, which needs further study. The relative content of *Asteroleplasma* was elevated for goats fed diets with 10% ramie. Zhan et al. (2017) reported that alfalfa flavonoids extract inhibited the proportion of *Asteroleplasma* in the rumen of dairy cow, but the function of *Asteroleplasma* was rather vague. Interestingly, 40% ramie proportion increased the concentration of ruminal acetate, butyrate, and total SCFA, but did not influence the composition of the rumen microbiota at genus level. The divergency of the results may signify that some other species could play an important role in the formation of acetate and butyrate. It was noted that a considerable amount of sequences was not classified into any genus, so it is difficult to relate these sequences with the activity of ruminal fermentation.

## 5 CONCLUSION

In conclusion, increasing the dietary proportion of ramie from 0% to 40% did not adversely affect the ruminal fermentation properties of goats, and 40% ramie increased the concentration of ruminal butyrate. The composition and the relative abundances of most ruminal microbiota remain unchanged with increasing ramie levels. The result of this study demonstrated that up to 40% ramie had no impairment in the ruminal fermentation or rumen microbiota. Considering the decreased daily feed intake when 40% ramie was included, we could expect better application effects of ramie at high levels by taking certain measures to improve the palatability of ramie for goats.

## CONFLICT OF INTEREST

None.

## ETHICAL APPROVAL

All experiments involving animals were conducted according to the principles of the Animal Care and Use Committee of the Hubei Academy of Agricultural Sciences (Hubei, China), which approved the study protocols.
